# Epitope-based universal vaccine for Human T-lymphotropic virus-1 (HTLV-1)

**DOI:** 10.1371/journal.pone.0248001

**Published:** 2021-04-02

**Authors:** Md. Thosif Raza, Shagufta Mizan, Farhana Yasmin, Al-Shahriar Akash, Shah Md. Shahik

**Affiliations:** 1 Faculty of Biological Sciences, Department of Genetic Engineering and Biotechnology, University of Chittagong, Chattogram, Bangladesh; 2 Bioinformatics Division, Disease Biology and Molecular Epidemiology Research Group, Chattogram, Bangladesh; Centers for Disease Control and Prevention, UNITED STATES

## Abstract

Human T-cell leukemia virus type 1 (HTLV-1) was the first oncogenic human retrovirus identified in humans which infects at least 10–15 million people worldwide. Large HTLV-1 endemic areas exist in Southern Japan, the Caribbean, Central and South America, the Middle East, Melanesia, and equatorial regions of Africa. HTLV-1 TAX viral protein is thought to play a critical role in HTLV-1 associated diseases. We have used numerous bio-informatics and immuno-informatics implements comprising sequence and construction tools for the construction of a 3D model and epitope prediction for HTLV-1 Tax viral protein. The conformational linear B-cell and T-cell epitopes for HTLV-1 TAX viral protein have been predicted for their possible collective use as vaccine candidates. Based on in silico investigation two B cell epitopes, KEADDNDHEPQISPGGLEPPSEKHFR and DGTPMISGPCPKDGQPS spanning from 324–349 and 252–268 respectively; and T cell epitopes, LLFGYPVYV, ITWPLLPHV and GLLPFHSTL ranging from 11–19, 163–171 and 233–241 were found most antigenic and immunogenic epitopes. Among different vaccine constructs generated by different combinations of these epitopes our predicted vaccine construct was found to be most antigenic with a score of 0.57. T cell epitopes interacted strongly with HLA-A*0201 suggesting a significant immune response evoked by these epitopes. Molecular docking study also showed a high binding affinity of the vaccine construct for TLR4. The study was carried out to predict antigenic determinants of the Tax protein along with the 3D protein modeling. The study revealed a potential multi epitope vaccine that can raise the desired immune response against HTLV-1 and be useful in developing effective vaccines against Human T-lymphotropic virus.

## Introduction

Human T Lymphocyte Virus 1 or HTLV-1 is a type C retrovirus that possesses proteins capable of oncogenesis [1]. Belonging to the *Retroviridae* family (sub-family: *Orthoretrovirinae*; genus: Deltaretrovirus), HTLV-1 has been identified to be the first human virus with oncogenicity [2]. Suspected of zoonotic origin, Human T Lymphocytic Viruses (HTLVs) come from a larger group of Primate T-lymphotropic viruses (PTLVs); their cousins being the Simian T-lymphotropic Viruses (STLVs) found in non-human primates (NHPs) [3–11]. To date, 4 types of HTLV viruses have been identified (HTLV-1, HTLV-2, HTLV-3, and HTLV-4) among which HTLV-1 is the most clinically active member [12]. According to a study in 2010, HTLV-1 infects approximately 20 million people globally [13]. Possible modes of transmission include breastfeeding (predominant), sexual intercourse, transfusion of infected blood components and sharing of needles and syringes [14]. Though the maximum of infected cases are asymptomatic, these individuals can still act as “Carriers” of the virus and are capable of transmitting the virus to a healthy individual [14].

HTLV-1 has etiological manifestation with severe inflammatory diseases like HTLV-1-Associated Myelopathy/Tropical Spastic Paraparesis (HAM/TSP), HTLV-1 associated Uveitis [12,15] and a lymphoproliferative disorder called Adult T Cell Leukemia/Lymphoma (ATL) [16–19]. HTLV-1 infected individuals have been found to be susceptible to other neurological [20], pulmonary [21], ophthalmological [22], rheumatological [23] and urological [24] coinfections because HTLV-1 can cause suppression of the immune system [25].

Approximately 100nm in diameter, HTLV-1 is an enveloped virus. The viral matrix protein (MA) lines the inner membrane of the virion envelope, the structure which encompasses the viral capsid (CA). The tenants of the capsid include two strands of genomic RNA (identical) and three enzymes; Pro (functional protease), IN (integrase) and RT (reverse transcriptase) [2,26]. The viral pathogenesis begins by membrane fusion between HTLV-1 and the target CD4+ T cells [27]. Cellular attachment is facilitated by a type of glycosaminoglycan known as Heparan Sulfate Proteoglycan [28] which is a commonly expressed cell surface molecule in case of mammalian cells [29] Glucose Transporter 1 (GLUT1) and Neuropilin-1 (NRP1—receptor for semaphorin-3A and VEGF-A165) have also been observed to play roles in cellular attachment [30,31]. Once the HTLV-1 virus infects a cell, the progression of the infection occurs by cell-to-cell transmission. This transmission process involves the formation of a viral synapse at the site of contact between an HTLV-1 infected T cell and a healthy T cell by polarization of a microtubule organizing center (MTOC) at a junctional point between the two cells.

The single-stranded, complex RNA genome of an HTLV-1 virus encodes a range of structural proteins including Gag, Pro, Pol (polymerase) which have roles in assembling the virion and maturation of the virus; Env, which assists the entrance of the viral nucleic acid inside the host and host transformation [13,32,33]; along with two important regulatory proteins Rex (encoded from ORFIII) and Tax (encoded from ORFIV). Among the two regulatory proteins, the Rex protein acts as a post-transcriptional regulator, Tax has been found to show oncogenic abilities [34–38] and has crucial roles in regulating viral transcription and transformation of T cells mediated by HTLV-1 [39,40]. Accessory genes p12 and p30 and their protein products p8 and p13 are encoded by ORFI and ORFII function in initiating the viral infection and play roles in creating persistence of the virus in animal models [41–44].

When a Human T-cell leukemia virus type 1 enters the human body through any of the transmission modes including transfusion, breastfeeding and sexual intercourse [14], HTLV-1 viruses by the help of membrane fusion facilitated by the virus’s own membrane protein Env and the target cell’s receptors insert their nucleic acid inside target CD4+ T cells (these cells are more susceptible as HTLV1 targets) [43]. The genomic RNA is reversely transcribed to DNA by reverse transcriptase enzyme (RT); following steps involving transportation of the viral DNA to the host nucleus and integration of the viral DNA with the host DNA. This amalgamated structure consisting of both the host and the viral DNA is termed as Provirus. The process of integration is worthwhile for the HTLV-1 virus as its copies are being made in spite of the whole viral structure not being present in the host by the help of the host’s own replication mechanism. A protein known as the HBZ protein (HTLV-1 basic leucine zipper factor) is encoded by the antisense transcript of the proviral genome [27] which upon interaction with the Jun family of AP-1 transcription factors JunB, c-Jun, JunD [45–47], cAMP response element binding (CREB) and CREB binding protein (CBP)/p300 regulate both cellular and viral gene transcription [48–50] and also plays a vital role in the proliferation of infected T cells [51–53].

Although the HTLV1 mediated pathogenesis is the cumulative result of multiple types of proteins, Tax protein has been found to be a key trigger element of a nuance of cellular events like resistance to apoptosis, cell signaling, cell cycle regulation and interference with checkpoint control and inhibition of DNA repair through its transactivational properties [26]. In patients with HTLV-1-Associated Myelopathy/Tropical Spastic Paraparesis (HAM/TSP), Tax protein is expressed at an elevated level regardless of the proviral load and therefore can be considered as an overt marker for HAM/TSP prognosis and a target for the development of therapeutics [1]. Tax protein has also been found to play significant role as a leukemogen and has successfully managed to arrest apoptosis in T-lymphocytes *in vitro* and cause cancer in transgenic animals [54,55]. Findings have established the fact that in 60% of the leukemic cases, Tax expression is undetectable [56]. This could be due to the fact the high levels of Tax protein in an infected cell make them susceptible to be attacked by Cytotoxic T Cells (CTL) which could be a reason for depleted levels of Tax expression [57].

For more than two and a half decades, multiple studies have been conducted on understanding the pathogenesis of HTLV-1 in order to develop therapeutics and vaccines against the virus. A favourable target for previous approaches to developing HTLV-1 vaccines and therapeutics has been the envelope glycoprotein (Env) since it is important for creating the baseline for viral gene transmission to a non-infected cell through cell mediated fusion [58]. As for a non-structural protein, Tax can also be a promising target for its importance as a determining factor of viral persistence and pathogenesis [1,39,40,55] for playing role as a key transcriptional activator [39,40]. Asymptomatic carriers, if not at an elevated level, have Tax protein expressions in infected cells (Median probability Tax protein expression of an infected cell in an Asymptomatic Carrier is 28% [1]). The goal of the current study is to map small continuous antigenic amino acid sequences in the Tax protein (epitopes) and selection of the most tangible Tax protein epitope candidate for *in silico* vaccine development against HTLV-1 following the principles of comparative modeling, stereochemical and epitope conservation analysis. *In vivo*, epitopes are presented to T cells through Human Leukocyte Antigen (HLA) molecules and are also detected by B cell receptors. The identification of epitopes by both T cells and B cells is a forerunner for the development of adaptive immunity against a specific antigen which the current study aims to develop against HTLV-1.

It is natural to consider the envelope glycoprotein of HTLV to be a more favourable candidate for vaccines; firstly because the envelope glycoprotein plays an important role in initiating viral pathogenesis by acting as the anchoring protein between the virus and the receptor and secondly, because previous studies have found highly immunodominant epitopes in the glycoprotein that are likely to evoke the immune cells [59–61]. However, factors like conservancy of the glycoprotein as well as nature of the epitopes should also be taken into account. In a previous study conducted on the molecular characterization of HTLV-1 gp46 glycoprotein, minor sequence variations were observed across different geographical regions as well as clinical characterization of the samples [62]. Additionally, the envelope glycoprotein is sensitive to mutations and will render non-expressive and dysfunctional if it undergoes any kind of mutation [63]. In case of epitope mapping, often in protein sequences immunodominant epitopes coincide with neutralizing epitopes, which can lead to faulty and diminished levels of epitope identification by immune cells [64]. A similar scenario was observed for envelope glycoprotein gp46, when Palker et al.’s study found that the central neutralizing region of gp46 (190–209) and the C-terminal neutralizing epitope (296–312) as Desgranges et al. defined had immunodominant properties [65]. Moreover, a study conducted by Horal et al., highlighted overlapping immunodominant and neutralizing epitopes in the regions including 176–199, 190–212, 224–244, 240–262, and 292–314 [66]. These facts imply the chances of improper identification of epitopes by immune cells and subsequent failure of the vaccine itself to provoke proper immune response. The Tax protein on the other hand is predominantly expressed in infected cells as transcription activators as well as exosomes, even in the early stages, therefore identification of the epitopes by immune cells and building the adaptive immune system against HTLV will be a more agile process [67–69].

The approach of this study to designing a Tax protein epitope based vaccine against HTLV-1 *in silico* might prove to be effective in activating the acquired immune system and stimulating specific humoral and cell mediated immune response against HTLV-1 in healthy individuals; treating both symptomatic and asymptomatic individuals with HTLV-1 infection and in preventing the development of further neurological, pulmonary, ophthalmological, rheumatological and urological co-infections in infected individuals.

## Materials and methods

**Fig 1:**
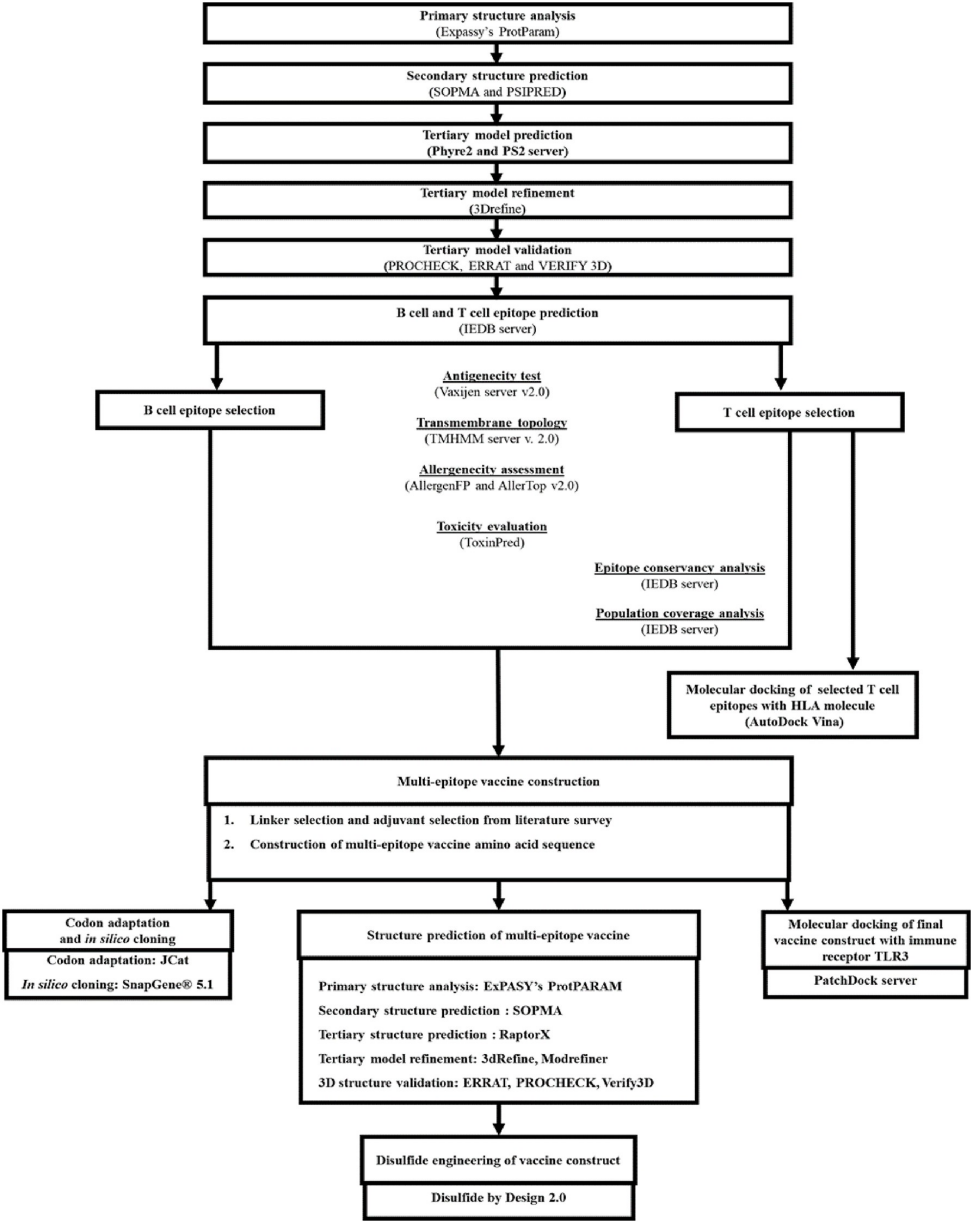
Working-flowchart of the multi-epitope vaccine construct design against HTLV-1.

A flow chart describing the overall procedures of construction of a multi-epitope based peptide vaccine for HTLV-1 Tax protein has been illustrated in Fig 1.

### Protein sequence retrieval

The amino acid sequence of Trans-activating transcriptional regulatory protein of HTLV-1 (Tax protein) was retrieved from the NCBI Protein database in FASTA format. The NCBI Protein database consists of a collection of sequences retrieved from SwissProt, PIR, PRF, PDB. It also includes translations from elucidated coding regions in GenBank, RefSeq and TPA. Numerous bioinformatics and immunoinformatics tools and databases were used to identify and analyse physicochemical, structural and functional properties of the selected Tax protein sequence and prediction of B-cell and T-cell epitopes.

### Primary and secondary structure analysis

The primary structure of the protein was evaluated using ProtParam tool of the Expasy server [70]. ProtParam computes various physical and chemical characteristics of given protein sequence, such as number of amino acid, isoelectric point (pI), molecular weight, instability index, aliphatic index, number of total atoms, grand average of hydropathicity (GRAVY). The secondary structure was analyzed using self-optimized prediction method with alignment (SOPMA) [71] and PSIPRED [72] to access properties like transmembrane helices, globular regions, bend region, random coil and coiled-coil region and to obtain a graphical presentation of the protein sequence of interest (HTLV-1 Tax protein).

### Tertiary structure analysis

The three dimensional structure from the amino acid sequence of the selected HTLV-1 Tax protein was predicted and analyzed using Phyre2 and (PS)^2^ Servers [73–75]. Phyre2 server allows 3D modelling of a provided amino acid sequence using the alignment of hidden Markov models by the help of HHsearch [76], improving accuracy of alignment and detection rate in a pronounced way. Phyre2 also models 3D structures for regions in a query sequence that does not have any detectable homology with recognized structures by incorporating an ab initio folding simulation called Poing [77]. The (PS)^2^ homology modelling server operates through a substitution matrix for detecting homologous proteins by combining both sequence and secondary structure information [74,75].

### Model refinement

The generated 3D model of HTLV-1 Tax protein was refined at an atomic level based on a reference 3D protein model using high resolution protein structure refinement tool ModRefiner [78] and 3DRefine [79].

### Validation of protein structure

Additional evaluation of the 3D structure was done by the help of multiple web tools. Ramachandran plot was generated using PROCHECK [80] to further assess the Phi/Psi angles for better understanding of the protein backbone confirmation. PROCHECK [80] was also used for stereochemical analysis of the predicted protein using a residue-by-residue approach. An additional evaluation of the generated 3D structure was carried out using ERRAT [81] and Verify 3D [82].

### B-cell epitope prediction

Linear B cell epitopes were predicted using sequential B-cell epitope predictor BepiPred-2.0 under IEDB (Immune Epitope Database) server [83]. Selection of the predicted B cell epitopes was done based on transmembrane topology (to discriminate between soluble and membrane proteins) and antigenicity for which TMHMM server 2.0 [84] and Vaxijen 2.0 web server [85] were used respectively. Allergenicity and toxigenicity of the selected epitopes were checked using AllerTOP v. 2.0 [86] and ToxinPred [87] respectively.

### T-cell epitope prediction

NetCTL 1.2 server was used to predict CTL epitopes for the selected HTLV-1 Tax protein sequence [88]. Binding affinity towards multiple HLA class molecules was assessed using MHC I binding prediction tools of IEDB server [89]. In case of predicting MHC class I binding epitopes, the Stabilized Matrix Base Method (SMM) was used in order to calculate the IC50 values [90]. Epitopes having an IC50 value less than 200nm were selected for the steps following.

### Population coverage calculation

Population coverage for the selected epitopes was observed using the IEDB resource for population coverage [91] considering denominated MHC restriction of T cell responses and polymorphic HLA combinations for different regions of the world. The selected epitopes were then scrutinized for their antigenic and immunogenic potentials using Vaxijen 2.0 [83] and immunogenicity analysis resources of the IEDB server [92]. The IEDB analysis resource was also used for assessing epitope conservancy. AllerTOP v. 2.0 [86] and ToxinPred [87] were used for assessing allergenic and toxigenic aptitude of the selected epitopes.

### 3D epitope structure prediction and molecular docking analysis

To substantiate the interaction between the selected T cell epitopes and HLA molecules, *in silico* docking was carried out between the molecules using AutoDock Vina [93]. For the process, HLA-A*0201 was selected due to having a high genetic frequency. In order to stimulate the docking, a crystal structure of HLA-A*0201 was retrieved from RCSB protein database (Research Collaboratory for Structural Bioinformatics) [94] having the PDB ID 1B0R. PEP-FOLD [95] server was used to predict peptide structures of the selected T cell epitopes based on antigenicity. To carry out docking simulation first of all the peptide ligand (Influenza Matrix Peptide) bound to HLA-A*0201 was removed by PyMol software package. This removed peptide ligand was then docked with the binding groove of HLA-A*0201. The binding groove had center box coordinates X: 29.900, Y: 0.614, Z: 49.512 and the dimensions of the grid box were X: 40, Y: 40 and Z: 40 (unit of the dimensions, Å) targeting the active site of the protein.

### Construction of multi epitope vaccine, modeling, and validation

The selected B cell and T cell epitopes based on antigenicity and allergenicity were linked in order to create a fusion peptide using GPGPG and AAY peptide linker [96] in multiple combinations. VaxinPad [97] was used to predict a peptide based adjuvant for the amino acid sequences generated using the selected epitopes. Selection of the adjuvant was based on immunomodulatory potential accompanied with properties like hydrophobicity, hydrophilicity, steric hindrance, solvation, hydropathicity and isoelectric point (pI). EAAK rigid peptide linker was used to link the selected adjuvant in the N-terminal end of the multi epitope combinations. The multi epitopes were then assessed for their antigenic and allergenic potencies using Vaxijen 2.0 web server [85] and AllerTOP v. 2.0 [86]. Multi epitope sequences exhibiting the maximum antigenic potential and non allergenicity were selected for tertiary structure evaluation. I-TASSER (Iterative Threading ASSEmbly Refinement) homology modelling tool was used to predict 3D structures [98–100] for the selected multi epitope sequences. The final 3D structures were further validated using PROCHECK [80], ERRAT [81] and Verify3D [82]. Upon completion of evaluation, the 3D structures were visualized using Pymol [101].

### Disulfide engineering of the 3D multi epitope vaccine constructs

To check if the structurally validated 3D vaccine constructs were accessible to the addition of novel disulfide bonds to provide the protein with increased stability and decreased conformational entropy [102], Disulfide by Design 2 (DbD2) was used for disulfide engineering of the 3D multi epitope vaccine constructs [102].

### Codon adaptation and *in silico* cloning

In order to express multi epitope vaccine construct selected based on antigenicity, peptide validation parameters and binding affinity towards HLA molecules in an *Escherichia coli* K12 strain codon optimization was done using Java Codon Adaptation Tool (JCat) [103]. The optimized codon sequence was further screened for expression parameters, codon adaptation index (CAI) and percentage of GC-content. For *in silico* cloning simulation, a bacterial, kanamycin resistant expression vector pETSUMO which consists of a 6x polyhistidine tag was selected [104]. The *in silico* restriction cloning simulation between the adapted codon sequence and pETSUMO expression vector was carried out using Snapgene software. The use of a pETSUMO expression vector will facilitate TA cloning as well as provide an easier screening modus operandi for transformed cells. The hexa histidine tag present in pETSUMO expression vector will facilitate swift detection of the recombinant vaccine construct in immunochromatographic assays [105].

### Molecular docking of vaccine constructs with TLR4

In order to predict the binding affinity between the Multi Epitope Vaccine Construct and Toll like receptor 4 [TLR-4 (PDB: 4G8A)], a member of the toll like receptor family of protein involved in triggering the innate immunity system [106], molecular docking approaches were implemented computationally. PatchDock (Beta 1.3 Version) docking server [107,108] was adopted to receptor-ligand docking and the generated Protein Data Bank (PDB) file of the protein-peptide docking complex was visualized in BIOVIA Discovery Studio Visualizer v12.1.0.15350.

## Results

### Sequence retrieval, transmembrane topology and antigenicity features analysis of the HTLV-1 Tax protein sequences

The complete amino acid sequences of HTLV-1 Tax protein were retrieved from NCBI database. Seven amino acid sequences of the protein were retrieved in FASTA format with accession number AYN25353.1, BBA30574.1, BBD74587.1, AYN25375.1, AYN25364.1, AYN25342.1, AYN25331.1 and BAX77785.1. Antigenicity of the protein was confirmed through assessment in VaxiJen v2.0 server and the Tax protein amino acid sequence with accession number BAX77785.1 was found to be most antigenic with a score of 0.4610 at threshold 0.4. Antigenicity is a prerequisite for a protein or amino acid sequence to be a vaccine candidate. The transmembrane topology of the protein was determined by TMHMM Server v. 2.0 and it was found that the protein fulfills the criteria of exo-membrane protein.

### Primary and secondary structure analysis

**Fig 2:**
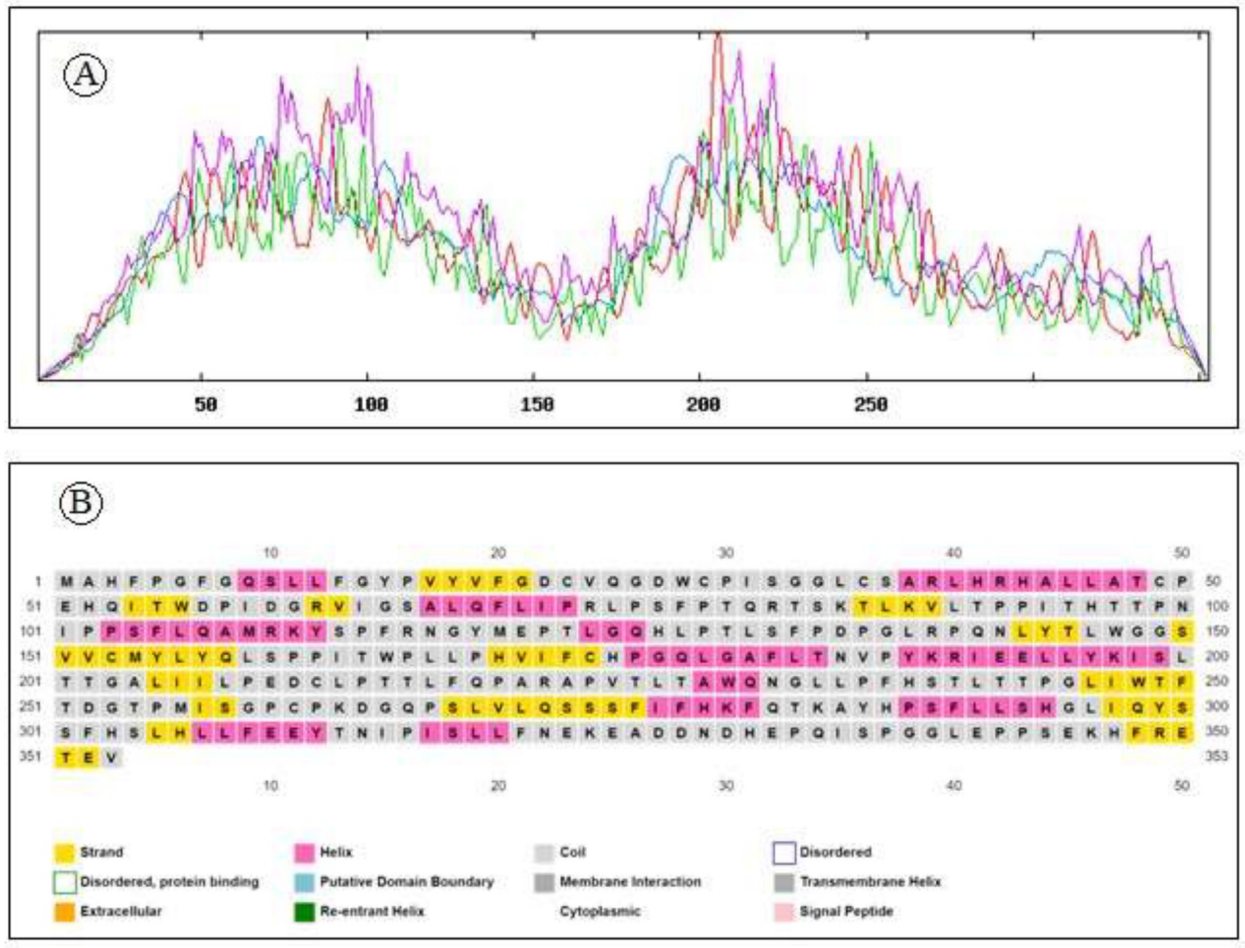
(a) Secondary structure plot of HTLV-1 Tax protein. Here, helix is indicated by blue, while extended strands and beta turns are indicated by red and green, respectively. (b) Distribution of different forms of secondary structure in HTLV-1 Tax protein.

A physico-chemical analysis of the protein primary structure was performed by the Expasy server’s ProtParam tool. From the result generated by ProtParam it was found that the 353 amino acids long HTLV-1 tax protein has a molecular weight of 39470.53Da with a high aliphatic index of 87.56%. Having pI of 6.45, less than 7, the protein belongs to negatively charged proteins. Instability index of 48.9 indicates that the protein is unstable *in vitro*. Interestingly it has a negative grand average hydropathy score accompanied with a high extinction coefficient as summarized in Table 1. Secondary structural features analysed by SOPMA and PSIPRED reveals the abundance of random coil (54.96%) followed by extended strand (21.53%), alpha helix (17.85%) and 5.67% of beta turn. in HTLV-1 Tax protein. The secondary structure plot is presented in Fig 2a while Fig 2b illustrates the distribution of different forms of secondary structure in HTLV-1 Tax protein.

**Table 1:** Different physio-chemical properties of HTLV-1 Tax protein.

Parameter	Value
Amino acids	353
Molecular weight	39470.53 Da
Theoretical isoelectric point (pI)	6.45
The instability index (II)	48.96
Grand average of hydropathicity (GRAVY)	-0.054
Total number of atoms	5551
Aliphatic index	87.56
No. of negatively charged residues (asp+glu)	25
No. of positively charged residues (arg+lys)	21

### Tertiary structure prediction, refinement and validation

**Fig 3:**
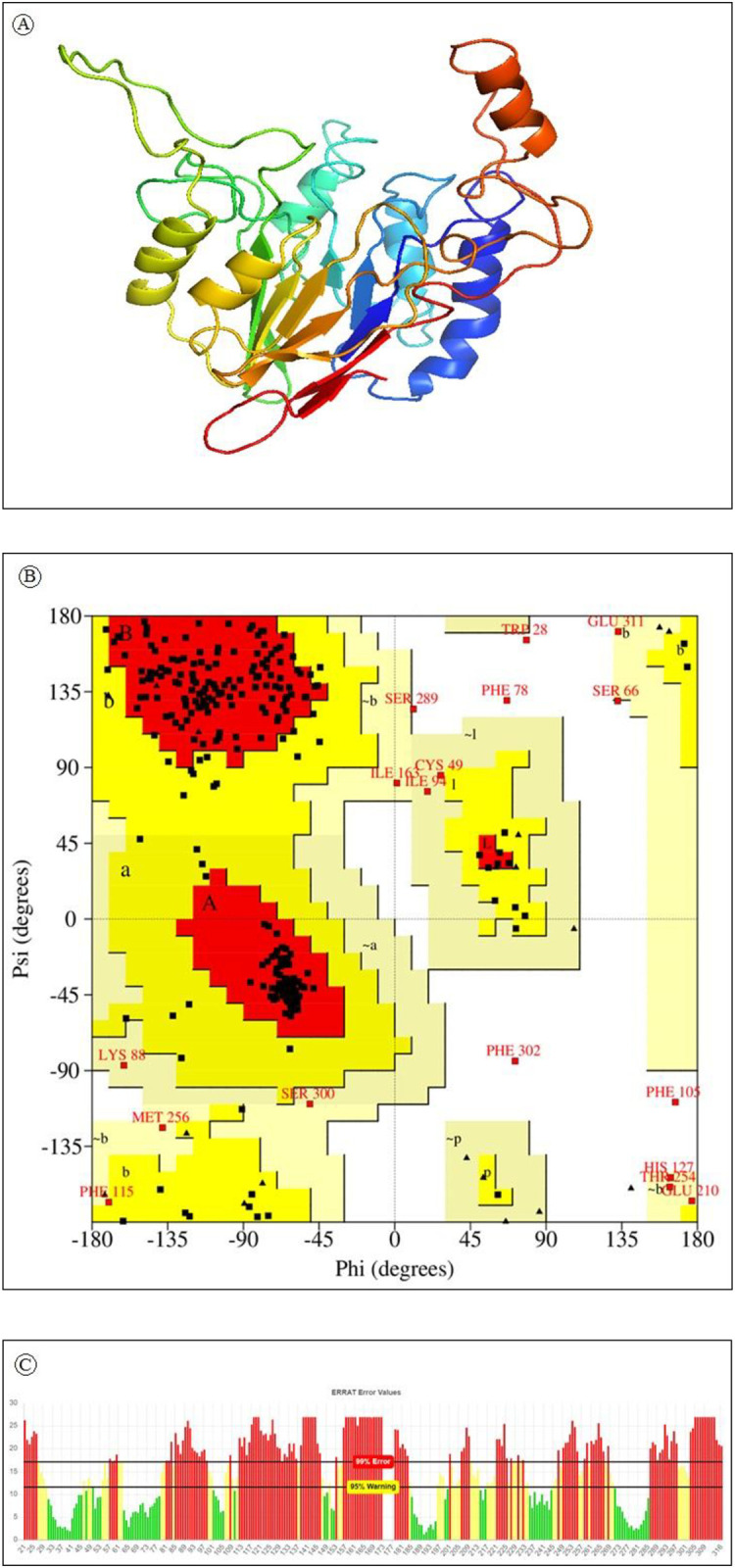
**a)** Predicted 3-dimensional structure of HTLV-1 Tax protein using comparative modeling. **b)** Analysis of Ramachandran plot of HLTV-1 Tax protein. Here, yellow indicates allowed region, red for favored region, light yellow shows generously allowed region while white for disallowed region. Torsion angels are determined by phi and psi angels. **c)** ERRAT generated result of Tax protein where 95% indicates rejection limit.

The tertiary structure of a protein is critical to its function and stability. 3D structure of the protein was predicted using the Phyre2 server and PS^2^ server. PDB ID 2I46 was selected as a template. The structure was predicted with 75% reliability. A pymol generated 3D structure of HTLV-1 Tax protein is displayed in Fig 3a. Major local distortions contained by homology based modeling include irregular H-hydrogen bonding networks, steric clashes and unphysical phi/psi angles which reduce the structure models usefulness for high-resolution functional analysis. Protein model refinement is required to overcome these distortions.

Refinement of the predicted 3D structure is required for bringing it closer to its native structure. 3Drefine was used for the structure refinement. The 3Drefine tool utilizes repetitive optimization of hydrogen bonding networks along with energy minimization at atomic-level on the optimized model by utilizing knowledge-based force fields and composite physics for efficient protein structure refinement. It generated 5 refined models with RMSD ranging from 0186 to 0.357. The best refined structure had the highest 3D score of 34961.1 accompanied by least RMSD 0.186.

After refinement the refined model was validated using PROCHECK, ERRAT and VERIFY 3D. Ramachandran plot generated by PROCHECK showed that 77.7 7% of the residues fall within the most favoured region (Fig 3b). Results generated from ERRAT showed an overall quality factor of 39.86 for the best refined structure (Fig 3c) and Verify3D depicts that only 27.20% of the residues in the protein had 3D-1D score equal or above 0.2.

### B cell epitope prediction

Overall antigenicity assessment of HTLV-1 tax protein by VaxiJen server regarded protein as a promising antigen at threshold 0.4. Transmembrane topology analysed by TMHMM predicted that all the residues of the protein are exposed outside the cell membrane. Epitope was predicted by the IEDB server using Kolaskar and Tongaonkar antigenicity and Bepipred linear epitope prediction method. Based on antigenicity test performed by VaxiJen server 7 epitopes (^324^KEADDNDHEPQISPGGLEPPSEKHFR^349^, ^252^DGTPMISGPCPKDGQPS^268^, ^114^PFRNGYMEPTLGQ^126^, ^51^EHQITWDPIDGR^62^, ^93^PITHTTPNIPPS^104^, ^131^LSFPDPGLRPQ^141^, ^77^SFPTQRTS^84^) were found to be antigenic (Table 2). TMHMM is used to evaluate the transmembrane topology of the antigenic B cell epitopes. EHQITWDPIDGR and SFPTQRTS with antigenicity score 1.3783 and 0.7813 respectively were exposed inside while epitope LSFPDPGLRPQ, PITHTTPNIPPS, PFRNGYMEPTLGQ, DGTPMISGPCPKDGQPS and KEADDNDHEPQISPGGLEPPSEKHFR having antigenicity score of 1.3581, 0.9582, 0.9083, 0.7207 and 0.7170 were exposed outside.

**Table 2:** Assessment of antigenicity score, transmembrane topology and toxicity of the antigenic B cell epitopes.

Start	End	Peptide	Length	Antigenecity	TMHMM	Toxicity
324	349	KEADDNDHEPQISPGGLEPPSEKHFR	26	0.7170	Outside	No
252	268	DGTPMISGPCPKDGQPS	17	0.7207	Outside	No
114	126	PFRNGYMEPTLGQ	13	0.9083	Outside	No
51	62	EHQITWDPIDGR	12	1.3783	Inside	No
93	104	PITHTTPNIPPS	12	0.9582	Outside	No
131	141	LSFPDPGLRPQ	11	1.3581	Outside	No
77	84	SFPTQRTS	8	0.7813	Inside	No

Toxicity of the antigenic epitopes was accomplished by the ToxinPred tool. All the examined epitopes reported to be non-toxic. Hydrophilic epitopes were subjected to assessment for electricity using AllerTopv2.0. KEADDNDHEPQISPGGLEPPSEKHFR and DGTPMISGPCPKDGQPS demonstrated no allergenicity while PFRNGYMEPTLGQ, PITHTTPNIPPS and LSFPDPGLRPQ were predicted as potential allergens as summarized in Table 4. Based on the antigenicity, surface accessibility and allergenicity 2 epitopes KEADDNDHEPQISPGGLEPPSEKHFR and DGTPMISGPCPKDGQPS spaning region 324–349 and 252–268 respectively were finally selected for vaccine model construction.

### T cell epitope prediction

NetCTL server and IEDB T cell epitope prediction tools were used to predict T cell epitopes of HTLV-1Tax protein. Among the 94 MHC class I epitopes predicted based on combined score 64 epitopes wear selected with IC50 value less than 200nm. 15 epitopes among them we are found to interact with multiple which were selected for antigenicity assessment at threshold level of 0.4. Epitopes with position 11–19, 151–159, 163–171, 178–186, 233–241, 297–305 and 307–315 were found antigenic (Table 3). LLFEEYTNI, QLGAFLTNV, LLFGYPVYV, ITWPLLPHV, GLLPFHSTL peptide sequence were found immunogenic when analysed with IEDB class I immunogenicity prediction tool. Immunogenic epitopes are listed in Table 4. From the transmembrane topology determination of these immunogenic epitopes by TMHMM server v. 2.0 it was found that all of these epitopes fulfills criteria of exomembrane protein.

**Table 3:** MHC class I epitopes based on their antigenicity, immunogenicity and epitope conservancy.

Epitopes	Antigenicity score	Immunogenicity score	Epitope conservancy (%)
LLFEEYTNI	0.4534	0.26	100
QLGAFLTNV	0.4664	0.18	100
LLFGYPVYV	0.4126	0.09	100
ITWPLLPHV	0.6704	0.05	100
GLLPFHSTL	0.9387	0.01	100
VVCMYLYQL	0.6350	-0.28	100
IQYSSFHSL	0.8634	-0.29	100

**Table 4:** Allergenicity, epitope conservancy and interacting MHC class I alleles for the immunogenic MHC class I epitopes.

Epitopes	Interacting Class I alleles (IC50<200)	Allergenicity	Epitope conservancy (%)
LLFEEYTNI	HLA-A*02:02, HLA-A*02:01, HLA-A*02:06, HLA-A*02:11, HLA-A*02:16, HLA-A*02:12, HLA-A*02:03, HLA-A*02:50, HLA-C*12:03, HLA-A*02:19, HLA-C*14:02, HLA-A*02:17.	Allergen	100
QLGAFLTNV	HLA-A*02:02, HLA-A*02:01, HLA-A*02:06, HLA-A*02:03, HLA-A*02:11, HLA-C*12:03, HLA-A*02:19, HLA-A*02:12, HLA-A*02:16.	Allergen	100
LLFGYPVYV	HLA-A*02:02, HLA-A*02:01, HLA-A*02:06, HLA-A*02:11, HLA-A*02:16, HLA-A*02:12, HLA-A*02:19, HLA-A*02:03, HLA-A*02:50, HLA-C*12:03, HLA-C*14:02, HLA-A*02:17, HLA-C*06:02, HLA-C*07:01.	Non-allergen	100
ITWPLLPHV	HLA-A*02:06, HLA-A*02:01, HLA-A*02:16, HLA-A*02:11, HLA-A*02:50, HLA-C*12:03, HLA-C*14:02.	Non-allergen	100
GLLPFHSTL	HLA-A*02:02, HLA-A*02:01, HLA-A*02:16, HLA-A*02:11, HLA-A*02:50, HLA-C*03:03, HLA-A*02:12, HLA-C*07:02, HLA-B*15:02, HLA-A*02:19, HLA-A*02:17, HLA-C*12:03, HLA-A*02:03.	Non-allergen	100

### Epitope conservancy, toxicity and allergenicity assessment

Epitope conservancy of the expected epitopes were tested by examining and matching all the epitopes obtained from HTLV-1 Tax protein. Epitope sequence LLFEEYTNI, QLGAFLTNV, LLFGYPVYV, ITWPLLPHV, GLLPFHSTL spaning 307–315,178–186, 11–19, 163–171, 233–241 position of HLTV-1 Tax were found to show 100% conservancy as predicted by the IEDB epitope conservancy prediction tool.

Toxicity was analysed by ToxinPred which indicated all 5 immunogenic epitopes as non toxic. The Allergenicity test carried out by AllerTop v2.0 depicted LLFGYPVYV, ITWPLLPHV and GLLPFHSTL as non allergen. These epitopes were also reported non toxic by ToxinPred (Table 4).

### Population coverage

**Fig 4:**
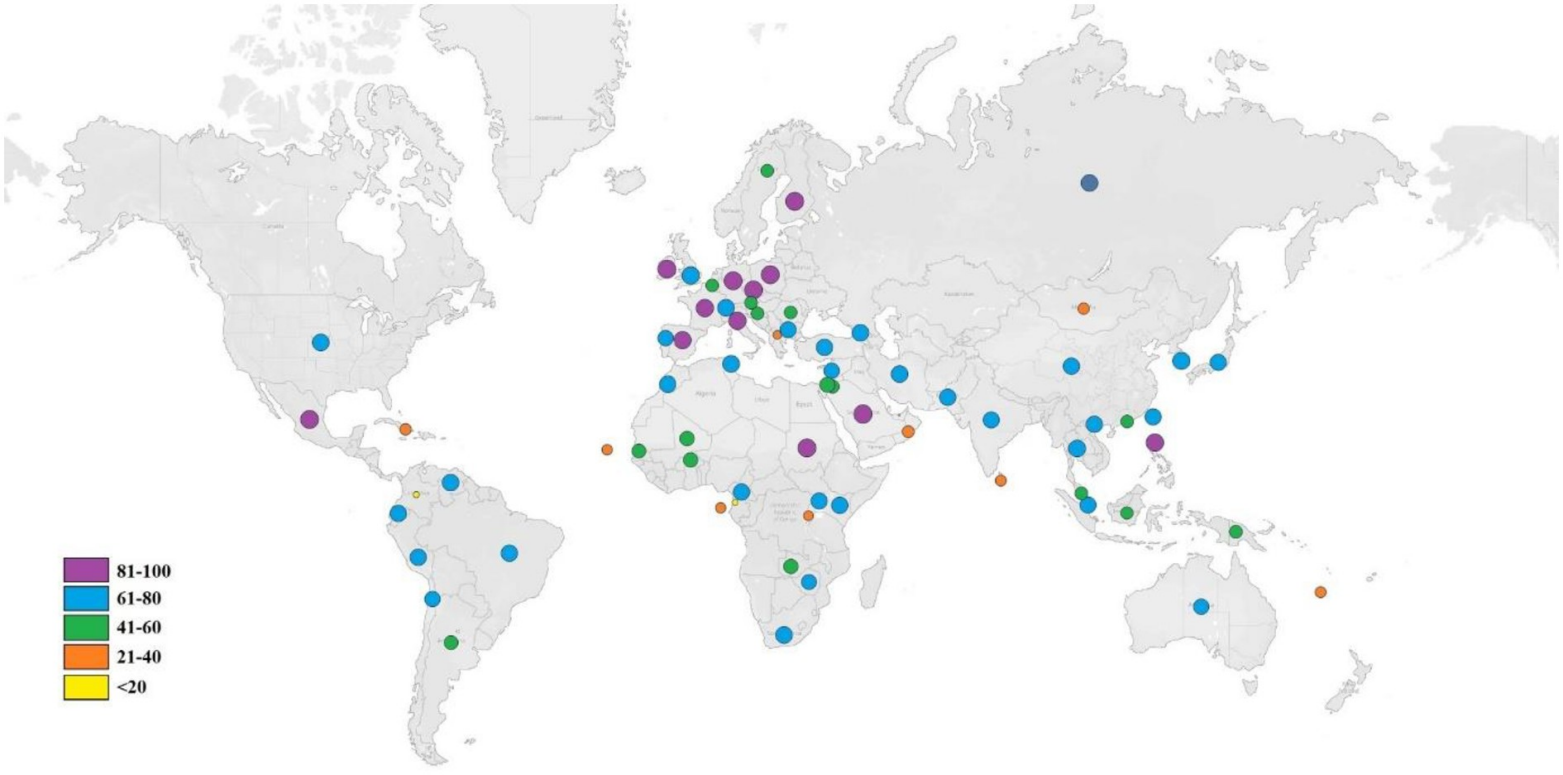
Worldwide population coverage of the selected for the multi-epitope vaccine. Global distributions of the selected epitopes are indicated by circles. Circle of different colors represents different ranges of population coverage.

As the different HLA alleles have different rates of occurrence in different ethnicities in the world, the analysis of individuals coverage by the respective HLA alleles of the predicted T-cell epitopes is an important part of an effective vaccine design. The population coverage analysis showed maximum coverage in Mexico (90.21%) followed by England (89.88%), South Africa (81.56%), North America (76.82%), South America (74.95%) and Japan (70.92%). Very low population coverage of 1.20% was observed in the United Arab of Emirates while it was more than 70% in three major parts of Asia, 70.48%, 71.78% and 74.53% respectively in Southeast Asia, Northeast Asia and East Asia. Cumulative population distribution of HLA alleles for the selected T cell epitopes is pictured by Tableau Public online public software in Fig 4.

### Molecular docking simulation of the HLA allele-peptide interaction

**Fig 5:**
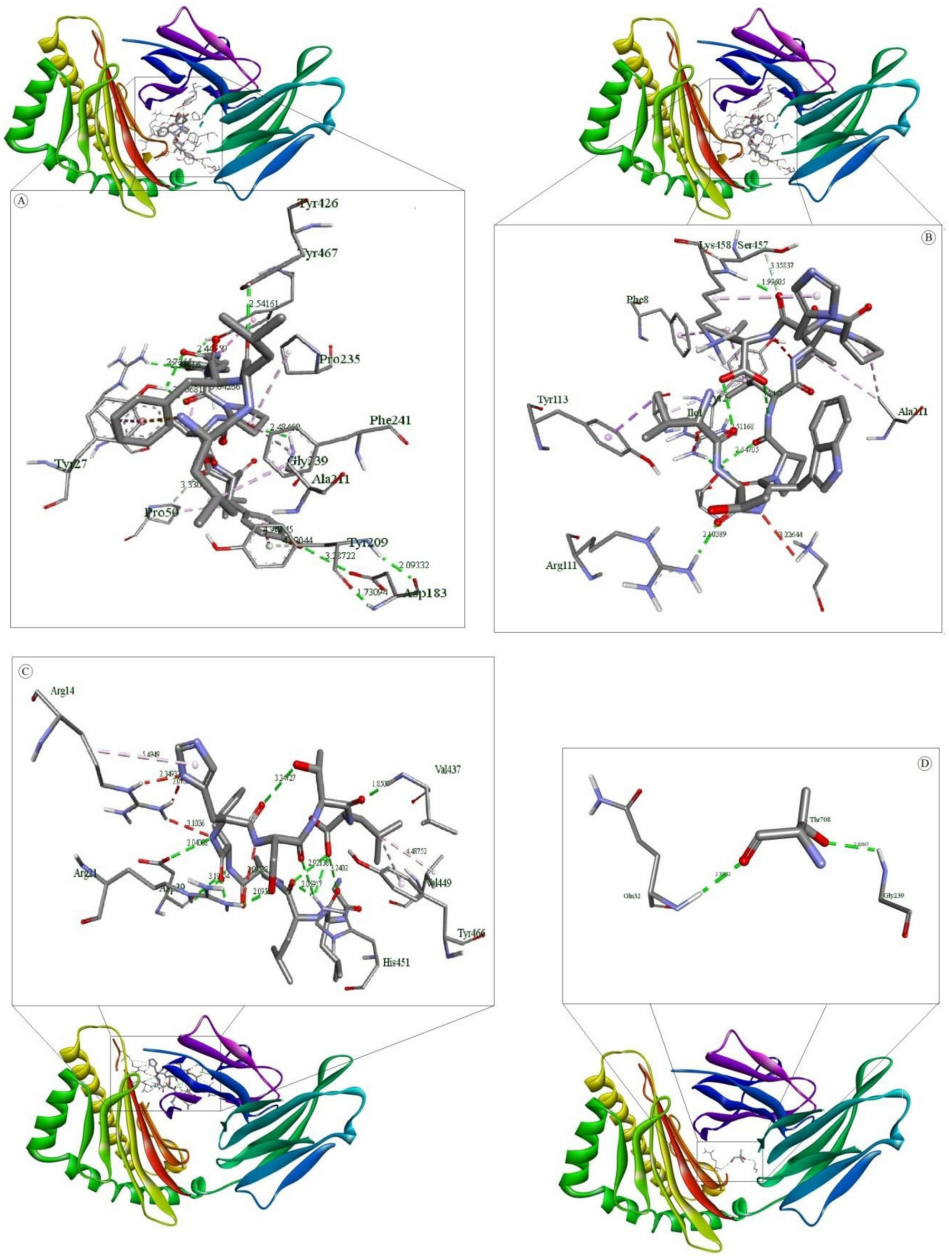
Molecular docking of selected T cell epitopes with HLA-A*0201. (A) shows interaction of LLFGYPVYV and HLA-A*0201 (B) shows interaction of ITWPLLPHV and HLA-A*0201 (C) shows interaction of GLLPFHSTL and HLA-A*0201 and (D) shows interaction of Influenza Matrix Peptide and HLA-A*0201.

Binding energy achieved for the peptide ligand (Influenza Matrix Peptide) was -5.7 Kcal/mol. A higher binding energy was acquired for our predicted epitopes ITWPLLPHV, LLFGYPVYV and GLLPFHSTL as follows -8.8, -8.6 and -8.5 Kcal/mol which suggest profound interaction between the predicted epitopes and HLA molecules. Analysis of the nonbonding interactions of the Influenza Matrix Peptide interacted with the active site A:TYR27, A:GLN32, A:PRO235, B:TYR426, B:SER452, B:ASP453, B:TYR463 and B:LEU465. T cell epitopes with the HLA-A*0201 reveals that the selected compounds interacted mostly with A:Try27, A:Pro235 and B:Tyr463 catalytic residues detected by Autodock Vina, as shown in Table 5 and Fig 5.

**Table 5:** Non-bonding interactions of three T-cell epitope ligands and an Influenza Matrix Peptide with HLA-A*0201.

Legends	Bonds [Donor. (Distance, Å). Acceptor] (Bond type)		
	Hydrogen Bond	Electrostatic Bond	Hydrophobic Bond
LLFGYPVYV	A:ARG6:HH11 (2.300):LEU2:O (HB) A:ARG6:HH12 (2.378):TYR5:OH (HB) A:TYR27:HH (2.187):VAL7:O (HB) A:GLN32:HN (2.147):VAL9:O1 (HB) A:GLY237:HN (2.983):TYR8:O (HB) A:GLY239:HN (2.319):TYR8:O (HB) B:LYS458:HN (2.966):LEU1:O (HB) B:TYR467:HH (2.328):TYR8:OH (HB) :VAL9:O1 (3.381) A:ASP30:O (HB) :TYR5:OH (3.319) A:ASP29:O (HB) A:ARG6:CD (3.297):TYR5:OH (CHB) A:THR31:CA (3.301):VAL9:O1 (CHB)	A:ASP30:OD2 (3.789):PHE3 (Pi-Anion)	:LEU1:CD2 (3.812) A:TYR113 (Pi-Sigma) B:TYR467 (5.311):TYR8 (Pi-Pi Stacked) A:ALA211 (4.904):PRO6 (A) A:PRO235 (4.589):PRO6 (A) A:PRO235 (4.365):VAL7 (A) B:LYS458 (4.867):LEU1 (A) B:LYS458 (4.180):LEU2 (A) :VAL7 (4.979) B:LEU465 (A) A:PHE241 (5.039):PRO6 (Pi-A) B:TYR426 (5.412):PRO6 (Pi-A) B:TYR463 (5.200):VAL7 (Pi-A)
ITWPLLPHV	A:ARG6:HH11 (2.792):HIS8:O (HB) A:TYR27:HH (2.216):TRP3:O (HB) A:GLN32:HN (2.281):THR2:OG1 (HB) A:GLU212:HN (2.545):VAL9:O2 (HB) B:TYR463:HH (2.132):LEU6:O (HB) :THR2:OG1 (3.371) A:ASP30:O (HB) :VAL9:N (3.296) A:ASP30:OD1 (HB) :VAL9:O2 (3.342) A:ASP30:OD1 (HB) A:ASP30:CA (3.338):LEU5:O (CHB) A:THR31:CA (3.542):THR2:OG1 (CHB)		B:TYR467 (5.495):TRP3 (Pi-Pi Stacked) :TRP3 (4.502) B:TYR467 (Pi-Pi Stacked) A:ALA49 (5.108):ILE1 (A) A:PRO50 (5.198):ILE1 (A) A:ALA211 (5.155):LEU5 (A) A:PRO235 (4.422):PRO4 (A) A:PRO235 (5.145):LEU5 (A) B:LYS458 (5.007):PRO7 (A) :PRO4 (5.006) B:LEU465 (A) A:TYR27 (5.433):LEU6 (Pi-A) A:PHE241 (5.371):LEU5 (Pi-A) B:TYR426 (5.493):PRO4 (Pi-A) B:TYR463 (4.805):PRO4 (Pi-A) B:TYR463 (5.333):LEU5 (Pi-A) :HIS8 (4.403) A:ALA211 (Pi-A)
GLLPFHSTL	A:GLY1:HT3 (2.059):LEU9:O2 (HB) A:SER2:HN (1.961):LEU9:O2 (HB) A:ARG6:HH12 (2.532):SER7:OG (HB) A:THR233:HN (2.685):LEU2:O (HB) A:THR233:HG1 (2.336):GLY1:O (HB) A:LYS243:HZ3 (2.532):GLY1:O (HB) B:SER457:HG (2.571):LEU3:O (HB) B:LYS458:HN (2.406):HIS6:NE2 (HB) :LEU3:N (3.215) A:GLU232:OE1 (HB) :LEU9:O1 (3.365) A:ASP29:OD1 (HB) :THR8:OG1 (3.190) A:ASP30:OD1 (HB) A:TYR27:HH (3.000):PHE5 (Pi-Donor HB) :SER7:OG (3.513):HIS6 (Pi-Donor HB)	:GLY1:N (3.609) A:GLU212:OE2 (Salt Bridge)	B:TYR463 (5.312):PHE5 (Pi-Pi Stacked) A:PRO210 (4.398):LEU9 (A) A:ALA211 (4.254):PRO4 (A) :PHE5 (4.024) A:PRO235 (Pi-A)
Influenza Matrix Peptide	A:TYR27:HH (1.971):ILE702:O (HB) A:GLN32:HN (2.086):GLY701:O (HB) B:SER452:HG (2.305):LEU703:O (HB)	:GLY701:N (5.208) B:ASP453:OD1 (Attractive Charge)	A:PRO235 (4.034):LEU703 (A) B:LEU465 (4.753):ILE702 (A) B:TYR426 (5.443):LEU703 (Pi-A) B:TYR463 (4.861):LEU703 (Pi-A)

(Pose predicted by AutoDock Vina where, HB = Conventional Hydrogen Bond, CHB = Carbon hydrogen bond, A = alkyl, Pi-A = Pi-Alkyl).

### Multi-epitope vaccine construction

**Fig 6:**
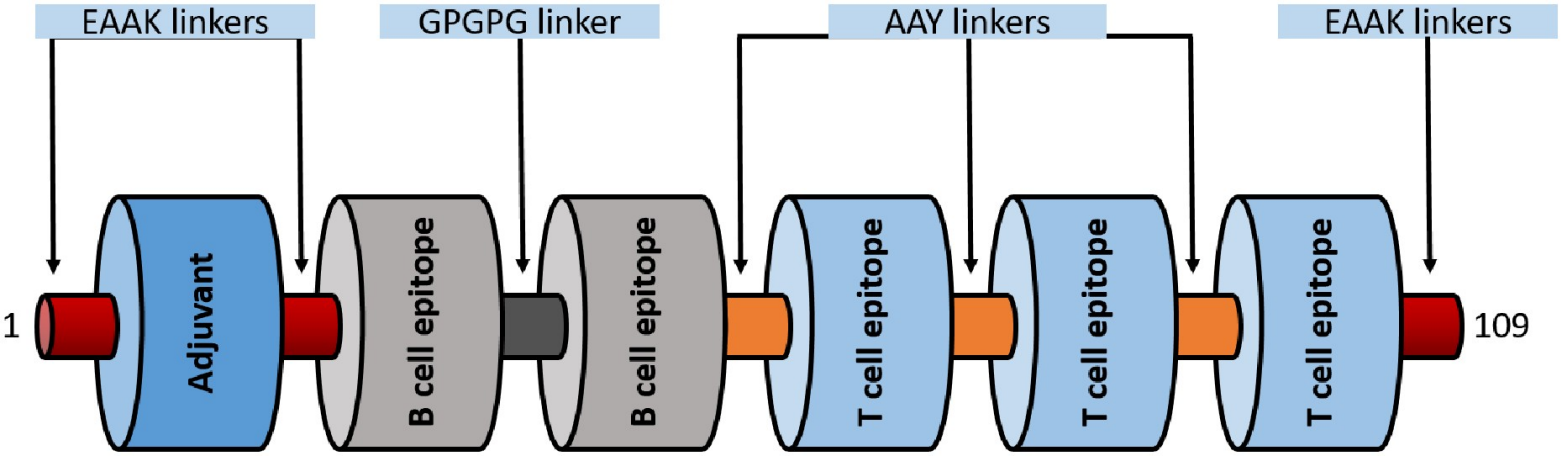
A diagrammatic representation of the final multi-epitope vaccine peptide. The 109-amino acid long peptide sequence containing an adjuvant (blue) at the N-terminal end linked with the multi-epitope sequence through an EAAAK linker (garnet). T cell epitopes are linked with the help of AAY linkers (orange) while the B cell epitopes are linked using GPGPG linkers (dark grey).

Three CTL epitopes (LLFGYPVYV, ITWPLLPHV and GLLPFHSTL) and two B cell epitopes (KEADDNDHEPQISPGGLEPPSEKHFR, DGTPMISGPCPKDGQPS). B cell epitopes were linked using GPGPG linkers while CTL epitopes by AAY linkers. A peptide adjuvant PMISWPCPKD was selected based on immunogenicity and high molecular weight using VaxinPred server. The Adjuvant was added to N termini of the vaccine construct using EAAAK linker. EAAAK linkers were used on both the terminals of the vaccine construct. Antigenicity score of the Multi-epitope vaccine construct was 0.57 as predicted by VaxiJen v2.0 server. A schematic diagram of 109 aa long multi-epitope vaccine is shown in Fig 6.

### Structure prediction of multi-epitope vaccine

Primary and secondary structure analysis provides insight about stability of a peptide. Various physicochemical parameters were determined by ExPASY’s ProtParam server. A high molecular weight of 11.523kDa is an indication of the antigenic nature of the vaccine construct. Theoretical pH is valuable in determining the buffer system for the vaccine purification process which was computed 5.18 representing the slightly acidic nature of the vaccine. Number of positively and negatively charged residues were found to be 8 and 13 respectively.

**Fig 7:**
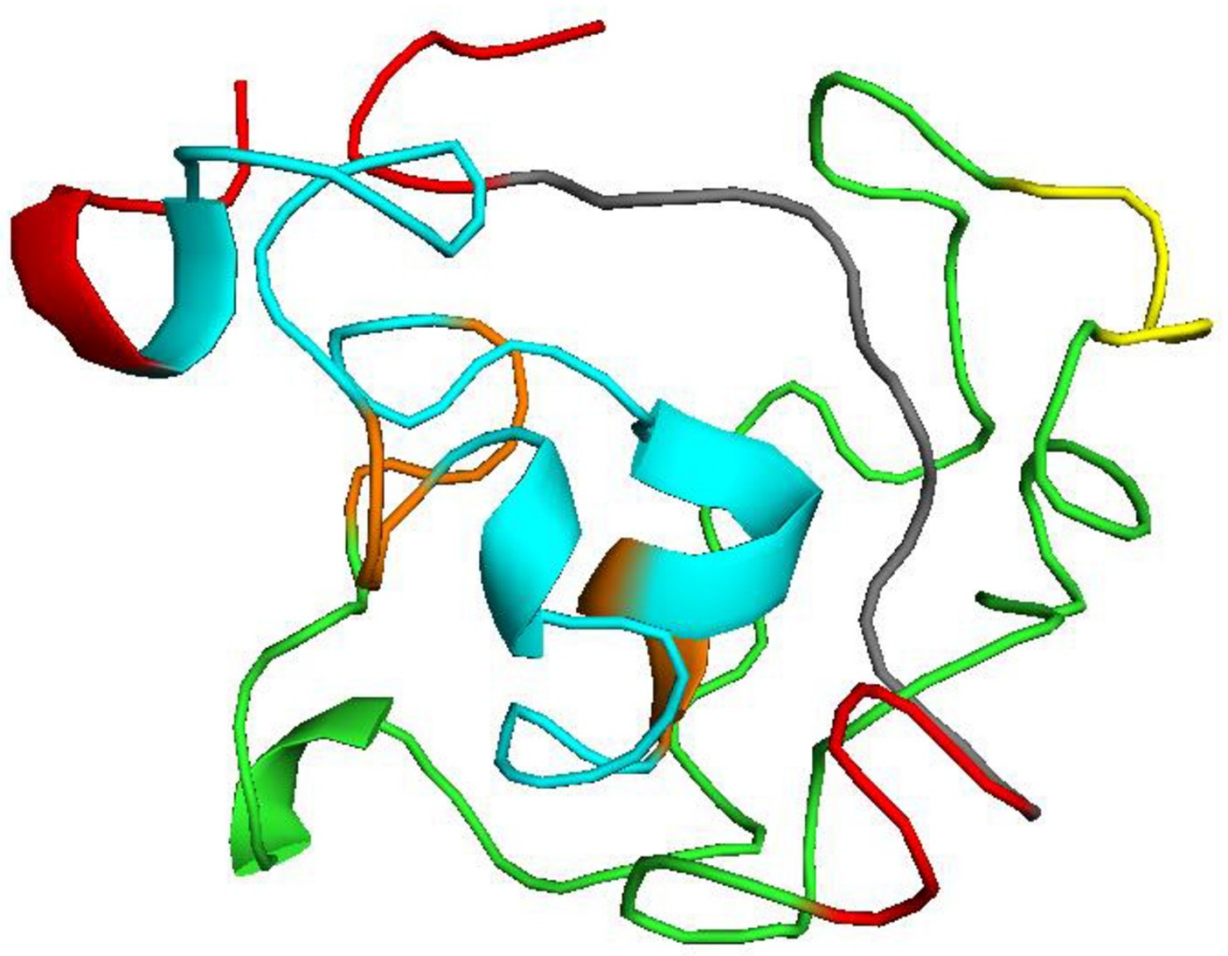
Tertiary structure of the multi-epitope vaccine. Red, orange and yellow color indicate EAAAK, AAY and GPGPG linkers respectively. B cell epitopes are shown in green while T epitopes in blue. A 10 mer adjuvant is visualized by gray color.

Assuming all cysteine residues are reduced, the extinction coefficient was 18450M-1cm−1 at 280 nm measured in water. The calculated half life was found 1 h in mammalian reticulocytes (*in vitro*), while >10 h in *E*. *coli* (*in vivo*) and 30 min in yeast (*in vivo*). The instability index was 38.16. Having an instability index below 40 classified the vaccine as stable. The aliphatic index was high and assessed to be 65.60 whereas the grand average of hydropathicity (GRAVY) was -0.367. A high aliphatic index supports the thermostability of the designed vaccine while a negative GRAVY score represents its hydrophilic nature. Secondary structure of the vaccine was assessed by SOPMA server using 109 aa long sequence. It predicted that 25.69%, 4.59%, 4.59% and 65.14% amino acids are involved in α-helix, extended strand, β-turn, and random coil, respectively. A probability score graph of frequency of helix, strand, turn, and coil at each amino acid position in the secondary structure of the final vaccine construct. Tertiary structure of the multi-epitope vaccine prediction by I-tasser which is an ordered approach for the prediction of protein structure and function. It identifies structural templates from the PDB by multiple threading approach LOMETS. Structure generated by the I-tasser is shown in Fig 7.

### Disulfide engineering, codon adaptation, and *in silico* cloning of the vaccine construct

Disulfide engineering was done through Disulfide by Design 2 (DbD2) which predicted a total of 20 pairs of residues for the probable disulfide bonds formation (Table 6) But, after considering the Chi3 value between −87 and +97 and the energy score less than 4 kcal/mol only 4 pairs of residue pairs were selected for making disulfide bonds.

**Table 6:** Possible disulfide bond between the residues of the vaccine construct.

Residue 1			Residue 2			Bond		
Chain	Seq	AA	Chain	Seq	AA	X_3_	kcal/mol	∑ B-factor
A	2	ALA	A	99	PRO	-67.47	5.50	5.17
A	2	ALA	A	101	LEU	+114.49	3.48	4.10
A	3	ALA	A	102	PRO	-73.27	3.51	4.13
A	4	ALA	A	99	PRO	-76.90	3.79	6.36
A	5	LYS	A	103	HIS	+81.03	3.37	6.01
A	13	PRO	A	16	GLU	+83.62	6.42	4.34
A	13	PRO	A	74	PHE	+122.24	1.95	4.61
A	24	PRO	A	26	ILE	+95.00	4.47	5.63
A	24	PRO	A	27	SER	+119.72	3.94	5.31
A	26	ILE	A	31	PRO	+118.95	4.74	4.57
A	27	SER	A	86	LEU	-61.55	3.42	4.60
A	28	GLY	A	51	GLU	-85.03	2.64	5.08
A	32	LYS	A	38	GLY	+114.69	1.93	6.06
A	52	PRO	A	55	SER	+123.75	6.02	5.12
A	70	ALA	A	74	PHE	+107.47	3.56	5.03
A	73	LEU	A	108	ALA	+78.29	5.00	4.98
A	77	PRO	A	104	VAL	+104.13	5.34	5.83
A	80	VAL	A	93	ALA	+87.23	6.55	5.90
A	94	ALA	A	98	TRP	+84.33	5.61	5.64
A	98	TRP	A	100	LEU	+120.55	7.58	4.13

JCat server was used for the adaptation of codon usage of the designed vaccine constructs for *E*. *coli* K12. An optimized codon sequence of 329 nucleotides was provided by JCat. The percentage of the GC-content of 50.78% and CAI of 1.0 of the optimized codon sequence ensured that the vaccine construct was highly expressed in *E*. *coli* K12. The improved DNA sequence was translated into our vaccine protein appropriately and GC-content increased to 58.10% for *E*. *coli* K12.

**Fig 8:**
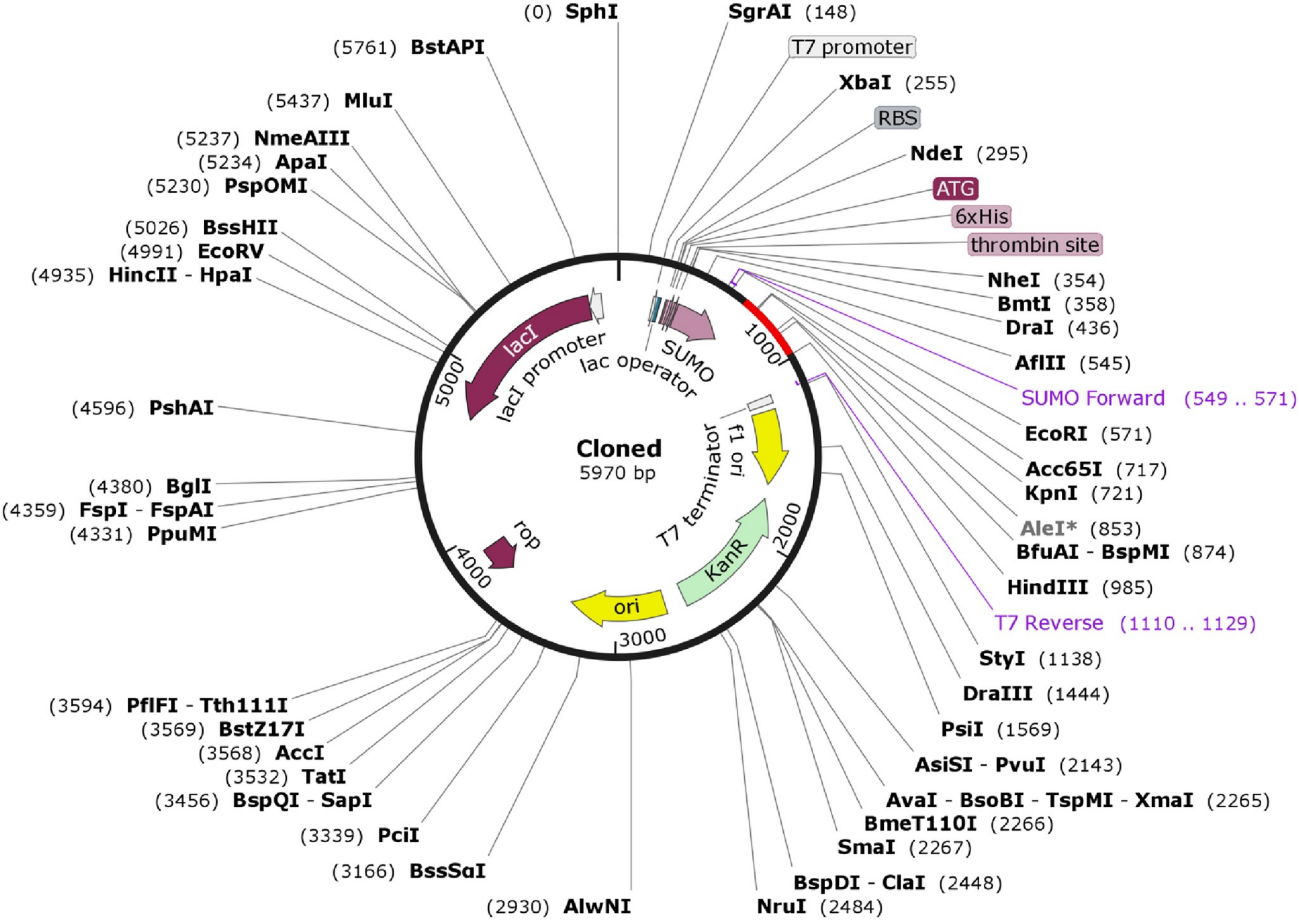
Cloning of the multi-epitope vaccine coding sequence in to pET-SUMO vector. Here the multi-epitope vaccine coding sequence of 329 bp is indicated by red color and pET-SUMO vector by black. A recombinant vector of 5970 bp is generated after insertion of the target nucleotide sequence.

Later on, the adapted codon sequence was cloned into the *E*. *coli* pET SUMO vector and *E*. *coli* strain DH5 alpha was selected as host. A 5970 bp recombinant vector was obtained after TA cloning of vaccine DNA sequence (Fig 8).

### Molecular docking of the multi-epitope vaccine with immune receptor *TLR4*

**Fig 9:**
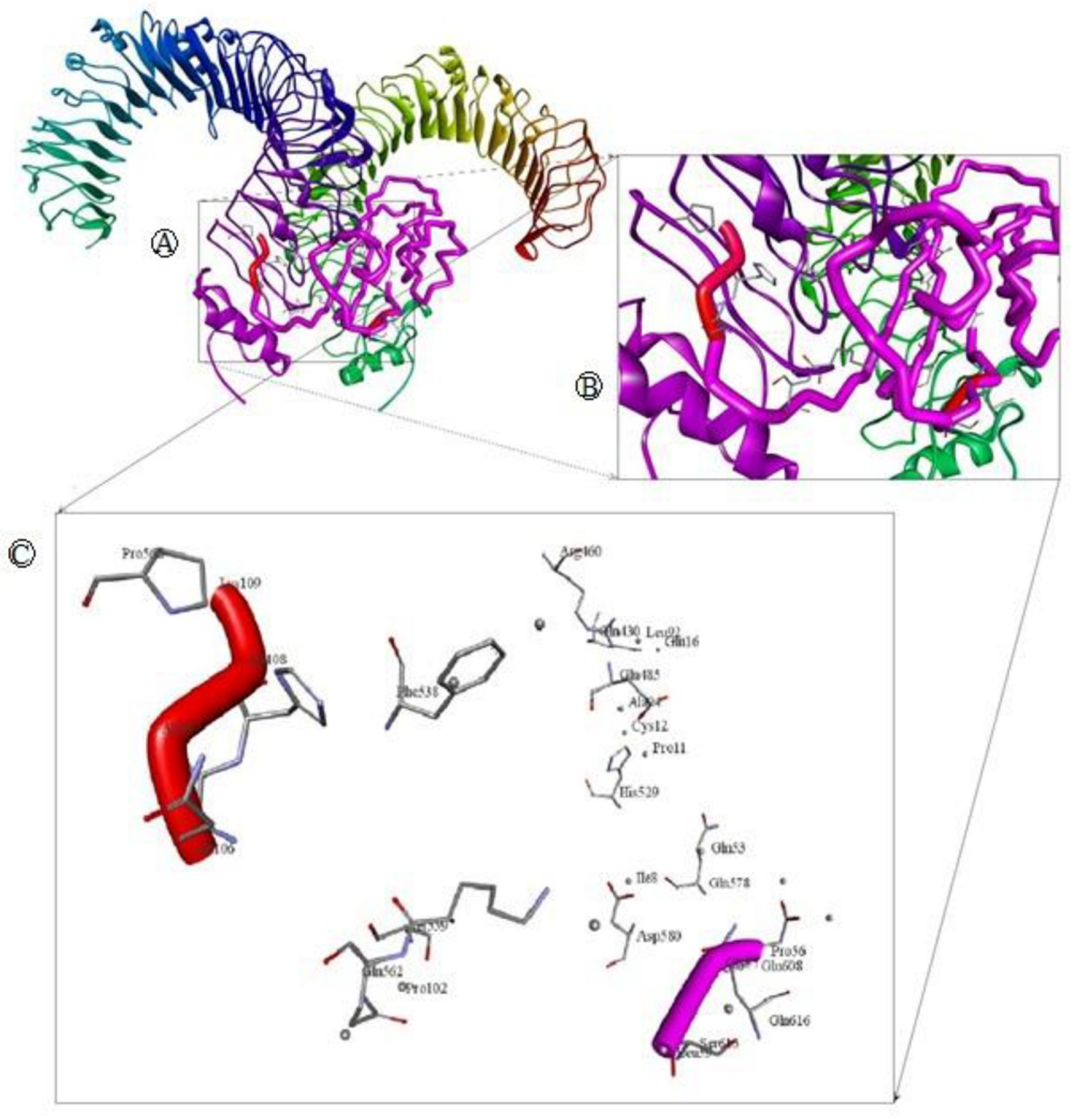
Molecular docking of multi epitope vaccine (MEV) with immune receptor (TLR4). (A) Whole Docked of MEV-TLR4 complex where MEV is purple and adjuvant colored red, chain A and B of TLR4 colored green and blue respectively. (B) Closer look of the main docking site. (C) Main interacting racedues and there interactions.

The docking of vaccine-receptor was performed using Patchdock server for evaluating the complex formation of the our vaccine construct with an immune receptor such as TLR4 and their binding affinity. The PatchDock server provided 20 docking complexes. Among the complexes, we selected only the docking complex with the highest negative Atomic Contact Energy (ACE) value for analysis. The ACE value of the selected docking complex was -247.59 which indicates spontaneous binding between the vaccine component and TLR-4. The selected docking complex is illustrated in Fig 9 along with molecular surface interaction as well as some bonding interactions. The elaborate interface residues between the vaccine component and TLR-4, bonding interactions and their distance are in the supplementary table (Table 1). The proper protein-protein docking between the vaccine component and TLR-4 will activate immune cascades for destroying the viral antigens 26.

## Discussion

HTLV-I is the prime causative agent leading to a disabling inflammatory disease HAM/TSP and an aggressive malignancy named Adult T cell Leukemia. To date no effective vaccine and treatment is available for HTLV-1 infection. Our study is focused on development of an effective vaccine against HTLV-1 Tax protein which plays a major role in viral pathogenesis. While the CTL response unique to Tax is a common occurrence in many HTLV-1-carriers, Tax protein is a major antigenic target for HTLV-1-specific CTLs [57]. Tax protein is the first HTLV-1 protein to be expressed in an infected cell [1]. It was reported as a potential immunotherapeutic target against HAM/TSP and ATL [109]. HBZ is another HTLV-1 protein which also plays an important role in viral infectivity surged with survival and growth of leukemic cells [110]. Despite the fact HBZ performs an essential role in the proliferation of HTLV-1-infected cells, it could additionally provide a unique mechanism that lets them evade immune recognition [111]. Antigenicity prediction of Tax and HBZ using VaxiJen server at threshold 0.4 regarded Tax protein as probable antigen with score of 0.461 while HBZ as non antigen with score of 0.3063. These contrasting facts show that Tax is a potential candidate for vaccine design albeit HBZ is a potential drug target.

A physico-chemical analysis of the protein sequence was done by the Expasy server’s ProtParam tool. It revealed an instability index of 48.9, which denotes, this protein will be unstable *in vitro* because a value over 40 is considered unstable [70]. Interestingly this protein was also predicted to have a high aliphatic index; it is the total volume occupied by aliphatic side chains and higher value is considered a positive factor for increased thermostability. Along with high extinction coefficient and negative GRAVY, the extents of other parameters imply the stability of the protein.

Results generated by secondary structure analysis tool PSIPRED and SOPMA showed the HTLV-1 Tax protein is adorned with 17.85% alpha helix and 54.96% random coils along with 5.26% extended strands and 21.53% beta turns. The abundance of coiled regions is an indication of higher conservation and stability of the model.

Tertiary structure of a protein determines its function and stability. To date no tertiary structure for HTLV-1 Tax is available in Protein Data Bank. We have generated a 3D structure of Tax protein using Phyre2 server and PS^2^ server with 75% reliability. Often 3D structures generated by bioinformatics tools contain significant local distortions, including unphysical phi/psi angles, and steric clashes irregular H-hydrogen bonding networks, which make the structure models unfit for high-resolution analysis of functions. Refinement of the modeled structures can bring up a solution of this problem [78,112]. Best model predicted by 3Drefine was selected after refinement based on having the highest 3D score and least RMSD. After refinement the protein model was validated by ERRAT, Verify3D, PROCHECK. ERRAT interpreted the overall quality of the model with the quality factor 78.313; this score represents the proportion of the protein that falls below the rejection limit of 95%. Verify 3D shows 63.49% of the residues have 3D-1D score above 0.2 whereas a good quality model required 80% of the residues to have 3D-1D> = 0.2. Ramachandran plot, a plot of the dihedral angles—phi (φ) and psi (ψ)—of the amino acids contained in a peptide, generated by PROCHECK illustrates that 75.7% of the amino acid fall within the most favored region and 17.4% in additional allowed region. Analysis based on 118 structures of resolution of minimum 2.0 Angstroms and R-factor less or equal to 20%, a good quality model should have over 90% of its residues in the most favoured regions.

Most B cell epitopes are discontinuous epitopes, epitopes composed of amino acid residues residing in different regions of the protein, which are arranged together by the folding of the protein chain [113,114]. These residue groups cannot be isolated in the same conformation from the antigen [114]. As per concern linear B cell epitopes for HTLV-1 tax are curated for IEDB. Bepipred linear epitope prediction method was used to predict 13 B cell epitopes. Among the predicted epitopes 7 of them exhibited antigenicity. From our data, we see that only 2 epitopes were present inside while other 5 epitopes were found outside. These 5 epitopes, KEADDNDHEPQISPGGLEPPSEKHFR, DGTPMISGPCPKDGQPS, PFRNGYMEPTLGQ, EHQITWDPIDGR, PITHTTPNIPPS, LSFPDPGLRPQ were assessed for toxicity and allergenicity which led as to select 2 non-allergenic and non-toxic B cell epitopes as potential vaccine candidate.

Once the proteins enter the host antigen-presenting cells (APC) of the, they are processed and then the T cell epitopes are proteolytically cleaved from the protein, and represented by the MHC molecules on the surface of APCs, exposing them to the receptors of T cells [114]. MHC class I molecules represent endogenous antigens such as intracellular bacterial, viral and tumor inducing proteins while MHC class II represent epitopes from the exogenous proteins. HTLV-1 Tax is preferentially produced endogenously in the cytoplasm of a host cell and localized in the cytoplasm with fewer speckle-like dots in the nucleus [115]. Induction of virus-specific CD8+ cytotoxic T lymphocytes (CTL) by MHC class I presented peptide is required for effective viral clearance [116]. MHC classI binding T cell epitopes were by NetCTL server and IEDB server. 94 MHC class I T cell epitopes were selected based on combined score from which 48 epitopes were extracted with IC50 less than 200nm. The lower the IC50 value the higher their binding affinity with the HLA molecules. About 16 epitopes interacted with more than 5 HLA alleles. Epitopes those which interacted with ≥5 MHC HLA-alleles are most likely to be potential vaccine candidates [117,118]. Antigenicity assessment with VaxiJen server at threshold 0.4 mined 7 epitopes to be antigenic and among them 5 were immunogenic. Epitope conservancy was found 100% for all 5 epitopes as predicted by IEDB server. Toxicity and allergenicity evaluation of the epitopes rendered all selected epitopes to be non-toxic but only 3 of them are non allergenic. Efficiency of a multiepitope vaccine greatly relies on precise interaction between epitopes and HLA alleles. MHC class I alleles having interaction with LLFGYPVYV, ITWPLLPHV and GLLPFHSTL were searched for population coverage. From our study the highest population coverage was recorded for Mexico (90.21%) followed by England (89.88%) and South Africa which is located in Sub Saharan Africa. South America and North America showed population coverage of 74.95% and 76.82% respectively while in Japan it was slightly lower (70.92%). East Asia, North East Asia and South Asia had population coverage just above 70%. Cameroon, a country of central Africa exhibited 71.39% population coverage while UNited Arab of Emirates was found with the lowest percentage (1.2%) of coverage. The major HTLV-1 highly endemic regions are the sub-Saharan Africa, South America Southwestern part of Japan, the Caribbean area, Australo-Melanesia and foci in the Middle East [119]. Age, ethnicity, mode of transmission are important factors influencing variation in population coverage [119].

Previous study of vaccine designing on HTLV-1 partially aligns with our study in that they have selected epitopes LLFGYPVYV, QLGAFLTNV and GLLPFHSTL as potential candidates for multivalent vaccine [l] but differ in that QLGAFLTNV was excluded from our vaccine as it was found allergenic. Instead, a non-allergenic T cell epitope ITWPLLPHV was assigned to be a part of our designed vaccine.

Docking simulation was carried out on AutoDock to assess the binding efficiency of the selected T cell epitopes to HLA molecules. The epitopes were docked with HLA-A*0201 complexed with a peptide with the carboxyl-terminal group substituted by a methyl group. The peptide had binding energy of -5.0Kcal/mol with the HLA molecules whereas our selected T cell epitopes ITWPLLPHV, LLFGYPVYV and GLLPFHSTL showed higher binding energy, -8.8 Kcal/mol, -8.6 Kcal/mol, -8.5 Kcal/mol respectively suggesting satisfactory binding accuracy of the predicted epitopes. The ITWPLLPHV is stabilized by twelve hydrogen bonds, eleven hydrophobic bonds and one electrostatic bond while interacting with the receptor protein. It also forms hydrogen bonds with catalytic residue A:Tyr27 and hydrophobic interaction with A:Pro235 and B:Tyr463. ITWPLLPHV interacts through ten hydrogen bonds with the catalytic residue A:Tyr27 and B:Tyr463 and fifteen hydrophobic interactions with the catalytic residue A:Pro235 and B:Tyr463. GLLPFHSTL forms thirteen hydrogen bonding interactions with catalytic residue A:Tyr27, one electrostatic and four hydrophobic interactions observed with the catalytic residue A:Pro235 and B:Tyr463. Two potential B cell epitopes and three T cell epitopes were selected for multi-epitope vaccine construction. A suitable adjuvant was selected using the VaxinPad tool based on antigenicity. B cell epitopes were linked using GPGPG linker while MHC class I epitopes were joined by AAY linker. EAAK linkers were added with N -termini and C -termini of vaccine construct. These linkers are widely used in multi-epitope vaccine construction [120–122]. Vaccine construct with highest antigenicity among multiple combinations of these epitopes and linkers was selected for further investigation. The vaccine construct was tested for allergenicity by AllerTop 2.0 and AllergenFP which declared the vaccine construct as non-allergen. An important insight was gained from the physicochemical properties and secondary structure analysis of the protein performed by ProtParam and SOPMA tools. It was found stable with instability index 30.57, negative GRAVY score (-0.367) and high aliphatic index are indications of the stability of the vaccine construct. Results generated by secondary structure prediction tool SOPMA showed the vaccine structure is dominated by 25.69% alpha helix and 65.14% random coils. The abundance of coiled regions indicates higher conservation and stability of the model [70,71,123]. As no suitable homologous template for homology modelling was not available for the vaccine construct peptide sequence ab initio modelling was executed using the I-Tasser tool to predict its 3D structure. Disulfide engineering was done at several positions on the vaccine tertiary structure. Disulfide bonds increase the stability of the protein [124].

Codon optimization was carried out in order to achieve high-level expression of our recombinant vaccine protein in *E*. *coli* (strain K12). Both the GC content (58.10%) and the codon adaptability index (1.0) were favourable for increased expression of the protein in bacteria. After codon adaptation the protein nucleic acid sequence was cloned in pET SUMO by TA cloning and expressed in *E*. *coli* strain DH5 alpha. DH5 alpha is a *E*. *coli* strain which allows easy selection of transformed colonies by blue-white screening. Again pET SUMO contains His Tag sequence at the 5’ end of the vaccine sequence which allows easy purification of the desired protein.

Moreover, it is necessary to know the immune response of TLR4 against the vaccine protein. TLR4 is known to activate innate immunity against HTLV-1 as reported in several studies [125,126]. It has been widely demonstrated that TLR4 plays an important role in the recognition of endogenous molecules which are released by necrotic cells and injured tissues [127]. These molecules activate an intense proinflammatory response through interaction with TLR4 [128]. To assess the binding affinity between the vaccine and TLR4 a molecular docking was performed and analysis of the result showed good binding affinity between them. A high binding affinity to TLR4 supports the acceptability of our predicted multi-epitope vaccine.

## Conclusion

Although the approaches in our study to predicting HTLV-1 TAX protein based epitopes and construction of a multi epitope vaccine were all in all *in silico*, the insights and outcome gained from the study can provide an elementary ground for expediting investigations related to designing and constructing epitope based vaccine against HTLV-1 in a wet lab and take it to *in vitro* studies from there. Further studies involving extensive laboratory assays and techniques might also render a higher a higher frequency and array of HLA molecules in terms of detecting the epitopes within the Tax protein.

## Supporting information

S1 Table Docking with TLR-4. (DOCX)

S2 Table T cell epitope. (PDF)

S3 Table B cell epitope. (PDF)
