# Supplementary material for: Epitope-based universal vaccine for Human T-lymphotropic virus-1 (HTLV-1)
Source: PLoS One. 2021 Apr 2;16(4):e0248001. doi: 10.1371/journal.pone.0248001 (PMC8018625; doi:10.1371/journal.pone.0248001)
Supplement: S1 Table — (DOCX) [file pone.0248001.s001.docx]

**Table S1:** Docking with TLR-4

| Protein | Legends | Atomic Contact Energy (ACE) | Amino acid residue involved in bond formation | | | TLR-4 Residues forming Bonds with  MEV | MEV Residues forming Bonds with TLR-4 |
| --- | --- | --- | --- | --- | --- | --- | --- |
|  |  |  | Donor | Acceptor | Bond Distance (Å) |  |  |
| TLR-4 | Multi Epitope Vaccine (MEV) | -247.59 | A:GLN430:OE1  A:HIS529:ND1  A:HIS529:CE1  A:HIS529:CE1  A:HIS529:HE1  A:GLN578:CG  A:ASP580:CG  A:ASP580:OD1  A:GLU608:CD  A:GLU608:OE1  A:GLU608:OE2  A:SER613:O  A:SER613:CB  A:SER613:OG  A:GLN616:CG  A:GLN616:CD  A:GLN616:OE1  A:GLN616:NE2  B:ARG460:NH2  B:GLU485:OE1  B:PHE538:CG  B:PHE538:CD1  B:PHE538:CE1  B:PHE538:CE2  B:PHE538:CZ  B:PHE538:CZ  B:SER559:O  B:LYS561:NZ  B:LYS561:NZ  B:GLN562:CB  B:GLN562:CG  B:GLN562:CD  B:GLN562:CD  B:GLN562:OE1  B:GLN562:NE2  B:GLN565:N  B:GLN565:CA  B:GLN565:C  B:GLN565:O  B:GLN565:CB  B:GLN565:CG  B:HIS566:CA  B:HIS566:C  B:HIS566:O  B:HIS566:CB  B:PRO568:N  B:PRO568:CG  B:PRO568:CD | :GLU16:CA  :CYS12:CA  :PRO11:CA  :CYS12:CA  :PRO11:CA  :GLN53:CA  :ILE8:CA  :ILE8:CA  :ASP46:CA  :ASP46:CA  :ALA45:CA  :GLY58:CA  :LEU59:CA  :LEU59:CA  :PRO56:CA  :PRO56:CA  :GLY57:CA  :PRO56:CA  :LEU92:CA  :ALA94:CA  :LYS65:CA  :LYS65:CA  :LYS65:CA  :HIS66:CA  :LYS65:CA  :HIS66:CA  :PRO102:CA  :PRO62:CA  :SER63:CA  :HIS103:CA  :HIS103:CA  :PRO102:CA  :HIS103:CA  :PRO102:CA  :PRO102:CA  :ALA106:CA  :ALA106:CA  :ALA107:CA  :ALA107:CA  :ALA106:CA  :ALA106:CA  :ALA108:CA  :ALA108:CA  :ALA108:CA  :ALA108:CA  :LYS109:CA  :LYS109:CA  :LYS109:CA | 1.92723  1.70717  1.97551  2.07227  1.81249  2.16818  2.04083  1.22279  2.28335  1.66895  2.06507  1.79082  2.20922  1.92647  1.90925  1.97728  2.10594  1.80292  2.08677  1.89978  1.82213  0.776672  0.976419  2.26044  2.00048  1.91703  1.81596  2.21082  2.27203  1.65492  0.415276  1.98426  1.83858  1.92654  1.68074  1.53257  1.11677  1.92518  1.36737  1.07187  2.17614  1.68514  1.31781  1.60342  2.13606  2.08387  2.25544  1.78624 | A:GLN430  A:HIS529  A:GLN578  A:ASP580  A:GLU608  A:SER613  A:GLN616  B:ARG460  B:GLU485  B:PHE538  B:SER559  B:LYS561  B:GLN562  B:GLN565  B:HIS566  B:PRO568 | ILE8  PRO11  CYS12  GLU16  ALA45  ASP46  GLN53  PRO56  GLY57  GLY58  LEU59  PRO62  SER63  LYS65  HIS66  LEU92  ALA94  PRO102  HIS103  ALA106  ALA107  ALA108  LYS109 |
