# Supplementary material for: Epitope-based universal vaccine for Human T-lymphotropic virus-1 (HTLV-1)
Source: PLoS One. 2021 Apr 2;16(4):e0248001. doi: 10.1371/journal.pone.0248001 (PMC8018625; doi:10.1371/journal.pone.0248001)
Supplement: S2 Table — (PDF) [file pone.0248001.s002.pdf]

Minimal Data Set

Table S2: T cell epitope

MHC class I epitopes with IC50<200					
**allel**	**start**	**end**	**epitope**	**IC50**	**rank**
HLA-A*02:02	11	19	LLFGYPVYV	2.089344239	0.2
HLA-A*02:01	11	19	LLFGYPVYV	2.444049514	0.2
HLA-A*02:06	297	305	IQYSSFHSL	7.15401753	0.3
HLA-A*02:06	163	171	ITWPLLPHV	7.422496475	0.3
HLA-A*02:01	155	163	YLYQLSPPI	7.693252737	0.2
HLA-B*08:01	37	45	SARLHRHAL	9.301435953	0.2
HLA-A*02:06	70	78	FLIPRLPSF	10.19882172	0.4
HLA-B*07:02	37	45	SARLHRHAL	12.18680887	0.2
HLA-A*02:02	178	186	QLGAFLTNV	13.80416049	0.5
HLA-A*02:06	177	185	GQLGAFLTN	22.51904662	0.8
HLA-A*02:02	307	315	LLFEEYTNI	23.87866265	0.9
HLA-A*02:02	155	163	YLYQLSPPI	24.49119634	0.9
HLA-A*02:06	11	19	LLFGYPVYV	24.80561506	0.8
HLA-A*02:06	155	163	YLYQLSPPI	26.27544016	0.9
HLA-B*07:02	72	80	IPRLPSFPT	32.80122022	0.3
HLA-A*02:01	163	171	ITWPLLPHV	39.45572246	0.7
HLA-A*02:02	70	78	FLIPRLPSF	54.07667744	1.9
HLA-B*08:01	107	115	QAMRKYSPF	54.89717547	0.2
HLA-B*39:01	297	305	IQYSSFHSL	55.00472529	0.4
HLA-B*58:01	222	230	RAPVTLTAW	56.2729911	0.5
HLA-A*02:06	144	152	YTLWGGSVV	61.31263769	2
HLA-B*58:01	284	292	KAYHPSFLL	64.16526615	0.5
HLA-B*07:02	175	183	HPGQLGAFL	66.81746579	0.5
HLA-A*02:02	67	75	ALQFLIPRL	68.39273951	2.4
HLA-B*58:01	198	206	ISLTTGALI	72.32692478	0.5
HLA-A*02:06	218	226	FQPARAPVT	75.08444933	2.5

HLA-B*58:01	62	70	RVIGSALQF	77.32145183	0.6
HLA-A*02:01	307	315	LLFEEYTNI	78.00276543	1.4
HLA-B*39:01	219	227	QPARAPVTL	79.14072054	0.5
HLA-A*02:01	178	186	QLGAFLTNV	79.45294511	1.4
HLA-A*02:06	281	289	FQTKAYHPS	81.76171642	2.7
HLA-A*02:01	67	75	ALQFLIPRL	84.54929682	1.4
HLA-B*07:02	219	227	QPARAPVTL	85.48501653	0.6
HLA-A*02:02	226	234	TLTAWQNGL	102.8039969	3.6
HLA-B*27:05	38	46	ARLHRHALL	104.6092287	0.4
HLA-A*02:01	70	78	FLIPRLPSF	106.4412583	1.7
HLA-A*03:01	276	284	FIFHKFQTK	114.2352141	0.5
HLA-B*08:01	70	78	FLIPRLPSF	114.4326618	0.4
HLA-A*02:02	297	305	IQYSSFHSL	115.6138863	3.9
HLA-A*02:06	159	167	LSPPITWPL	117.6386413	3.7
HLA-A*02:06	178	186	QLGAFLTNV	125.1843389	3.9
HLA-B*07:02	4	12	FPGFGQSLL	127.9057298	0.8
HLA-A*02:02	64	72	IGSALQFLI	127.9410763	4.2
HLA-A*02:02	233	241	GLLPFHSTL	128.8279215	4.2
HLA-A*02:02	13	21	FGYPVYVFG	129.1249005	4.2
HLA-A*02:02	151	159	VVCMYLYQL	129.4225642	4.3
HLA-B*07:02	186	194	VPYKRIEEL	129.6850757	0.8
HLA-A*02:01	233	241	GLLPFHSTL	131.8590675	2
HLA-B*07:02	101	109	IPPSFLQAM	134.8621255	0.8
HLA-A*02:06	13	21	FGYPVYVFG	135.3785942	4.2
HLA-A*02:02	158	166	QLSPPITWP	145.8847851	4.7
HLA-B*27:05	189	197	KRIEELLYK	147.7644631	0.5
HLA-A*02:02	300	308	SSFHSLHLL	149.9719367	4.8
HLA-A*02:02	123	131	TLGQHLPTL	151.35961	4.8
HLA-A*02:02	63	71	VIGSALQFL	153.4652319	4.9
HLA-A*02:06	230	238	WQNGLLPFH	156.8736495	4.8
HLA-A*02:02	159	167	LSPPITWPL	157.0398964	4.9

HLA-A*24:02	164	172	TWPLLPHVI	158.7193952	0.3
HLA-B*58:01	64	72	IGSALQFLI	161.9198154	1
HLA-A*02:06	284	292	KAYHPSFLL	162.760869	4.9
HLA-A*02:06	269	277	LVLQSSSFI	164.2668768	4.9
HLA-A*02:06	307	315	LLFEEYTNI	165.4055204	5
HLA-A*02:02	299	307	YSSFHSLHL	171.7947944	5.2
HLA-A*02:06	151	159	VVCMYLYQL	172.8024915	5.1
HLA-A*02:01	151	159	VVCMYLYQL	175.0289954	2.5
HLA-A*02:06	85	93	KTLKVLTPP	182.6206987	5.3
HLA-A*02:02	312	320	YTNIPISLL	184.5057903	5.4
HLA-A*02:02	127	135	HLPTLSFPD	189.6749595	5.5
HLA-A*02:02	130	138	TLSFPDPGL	191.4300003	5.6
HLA-B*07:02	261	269	CPKDGQPSL	193.5931561	1
HLA-A*02:01	297	305	IQYSSFHSL	196.3857628	2.7
HLA-A*02:06	271	279	LQSSSFIFH	197.9474904	5.7
HLA-A*02:06	9	17	QSLLFGYPV	198.4038065	5.7

MHC class I epitopes with IC50<200 interacting with multiple MHC class I allels			
**peptide**	**length**	**immunogenecity score**	**antigenecity**
LSPPITWPL	9	0.33184	0.3466 (Probable NON-ANTIGEN)
LLFEEYTNI	9	0.26175	0.4534 (Probable ANTIGEN)
ALQFLIPRL	9	0.21556	-0.3361 (Probable NON-ANTIGEN)
QLGAFLTNV	9	0.18291	0.4664 (Probable ANTIGEN)
QPARAPVTL	9	0.14996	0.2750 (Probable NON-ANTIGEN)
SARLHRHAL	9	0.13602	-0.0517 (Probable NON-ANTIGEN)
FGYPVYVFG	9	0.1276	-0.4248 (Probable NON-ANTIGEN)
LLFGYPVYV	9	0.09074	0.4126 (Probable ANTIGEN)
ITWPLLPHV	9	0.04904	0.6704 (Probable ANTIGEN)
GLLPFHSTL	9	0.01275	0.9387 (Probable ANTIGEN)
FLIPRLPSF	9	-0.03402	-0.0001 (Probable NON-ANTIGEN)
IGSALQFLI	9	-0.04185	0.2352 (Probable NON-ANTIGEN)
VVCMYLYQL	9	-0.27904	0.6350 (Probable ANTIGEN)
IQYSSFHSL	9	-0.28793	0.8634 (Probable ANTIGEN)
YLYQLSPPI	9	-0.30013	0.2734 (Probable NON-ANTIGEN)
