# Supplementary material for: Epitope-based universal vaccine for Human T-lymphotropic virus-1 (HTLV-1)
Source: PLoS One. 2021 Apr 2;16(4):e0248001. doi: 10.1371/journal.pone.0248001 (PMC8018625; doi:10.1371/journal.pone.0248001)
Supplement: S3 Table — (PDF) [file pone.0248001.s003.pdf]

Table S3: B cell epitope:

Antigenic B cell epitopes (Antigenecity score threshold 0.4)

Start	End	Peptide	Length	Antigenecity	TMHMM	Toxicity
324	349	KEADDNDHEPQISPGGLEPPSEKHFR	26	0.717	Outside	No
252	268	DGTPMISGPCPKDGQPS	17	0.7207	Outside	No
114	126	PFRNGYMEPTLGQ	13	0.9083	Outside	No
51	62	EHQITWDPIDGR	12	1.3783	Inside	No
93	104	PITHTTPNIPPS	12	0.9582	Outside	No
131	141	LSFPDPGLRPQ	11	1.3581	Outside	No
77	84	SFPTQRTS	8	0.7813	Inside	No

*Selected B cell epitopes are marked in highlighted in blue
